# Sarcopenia index based on serum creatinine and cystatin C is associated with mortality in middle-aged and older adults in Chinese: A retrospective cohort study from the China Health and Retirement Longitudinal Study

**DOI:** 10.3389/fpubh.2023.1122922

**Published:** 2023-03-21

**Authors:** Yang Wu, Hai Wang, Yingmu Tong, Xing Zhang, Yunxiang Long, Qinglin Li, Jie Ren, Chang Liu

**Affiliations:** ^1^Department of Hepatobiliary Surgery, The First Affiliated Hospital of Xi'an Jiaotong University, Xi'an, China; ^2^Department of General Surgery, The First Affiliated Hospital of Xi'an Jiaotong University, Xi'an, China; ^3^Department of Surgical ICU, The First Affiliated Hospital of Xi'an Jiaotong University, Xi'an, China

**Keywords:** sarcopenia, middle-aged, all-cause mortality, older adults, CHARLS

## Abstract

**Background:**

The sarcopenia index (SI, serum creatinine/serum cystatin C × 100) is recommended for predicting sarcopenia. There were several studies showing that lower SI is associated with poorer outcomes in the older adults. However, the cohorts studied in these researches were mainly patients hospitalized. The aim of this study was to evaluate the correlation between SI and all-cause mortality among middle-aged and older adults from the China Health and Retirement Longitudinal Study (CHARLS).

**Materials and methods:**

A total of 8,328 participants meeting the criteria were enrolled in this study from CHARLS between 2011 and 2012. SI was calculated as [serum creatinine (mg/dL)/cystatin C (mg/L)] × 100. Mann-Whitney *U*-test and Fisher's exact test were used to assess balance in baseline characteristics. Kaplan-Meier, log-rang analysis, univariate and multivariate Cox hazard ratio regression models were used to compare the mortality between different SI levels. The dose relationship between sarcopenia index and all-cause mortality was further assessed by the cubic spline functions and smooth curve fitting.

**Results:**

After adjustment for potential covariates, we found SI was significantly correlated with all-cause mortality [Hazard Ratio (HR) = 0.983, 95% confidence interval (CI) 0.977–0.988, *P* < 0.001]. Similarly, as SI was used as a categorical variable according to quartiles, higher SI was associated with lower mortality [Hazard Ratio (HR) = 0.44, 95% CI 0.34–0.57, *P* < 0.001] after adjustment for confounders.

**Conclusions:**

Lower sarcopenia index was associated with higher mortality among middle-aged and older adults in China.

## Introduction

Sarcopenia, which often exacerbates during the aging process, manifests as reduced skeletal muscle mass and weakness of mass strength and/or physical performance (1), Individuals diagnosed with sarcopenia are often associated with function decline, decreased quality of life, even increased risk of mortality (2–5). As the aging of the population poses considerable social challenges, the adverse effect bought by sarcopenia is becoming much more profound.

While several ways were recommended for assessing sarcopenia during hospitalization, the standard criteria for evaluating sarcopenia include low skeletal muscle mass (SMM) and low muscle function. The golden standard of SMM assessment is the medical imaging afforded by computed tomography (CT), magnetic resonance imaging (MRI), and so on. Besides these, low muscle function was evaluated by gait speed. All ways of assessment could be both time-consuming and expensive.

Recently, a novel sarcopenia index (SI) was developed by Kashani et al., which is calculated as [serum creatinine (mg/dL)/cystatin C (mg/dL)] × 100. The SI showed a positive relationship with muscle mass and strength. Besides these, this index also showed a good ability to diagnose sarcopenia in critically ill patients (6). There were also other studies that showed that low SI was associated with poor long-term prognosis in adult ICU patients and hospitalized older patients (6–9). However, most of these studies were based on patients treated in hospitals, while there is a little study investigating the relationship between SI and long-term prognosis among middle-aged and older adults people in the general person.

In this study, we aim at clarifying the relationship between SI and all-cause mortality using data from the China Health and Retirement Longitudinal Study (CHARLS), which was a nationally representative survey.

## Methods

### Study design and population

The cohort enrolled in this study was obtained from the China Health and Retirement Longitudinal Study (CHARLS), which is held by the National Development Institute of Peking University. More details about this cohort were described in other research papers (10).

**Figure 1:**
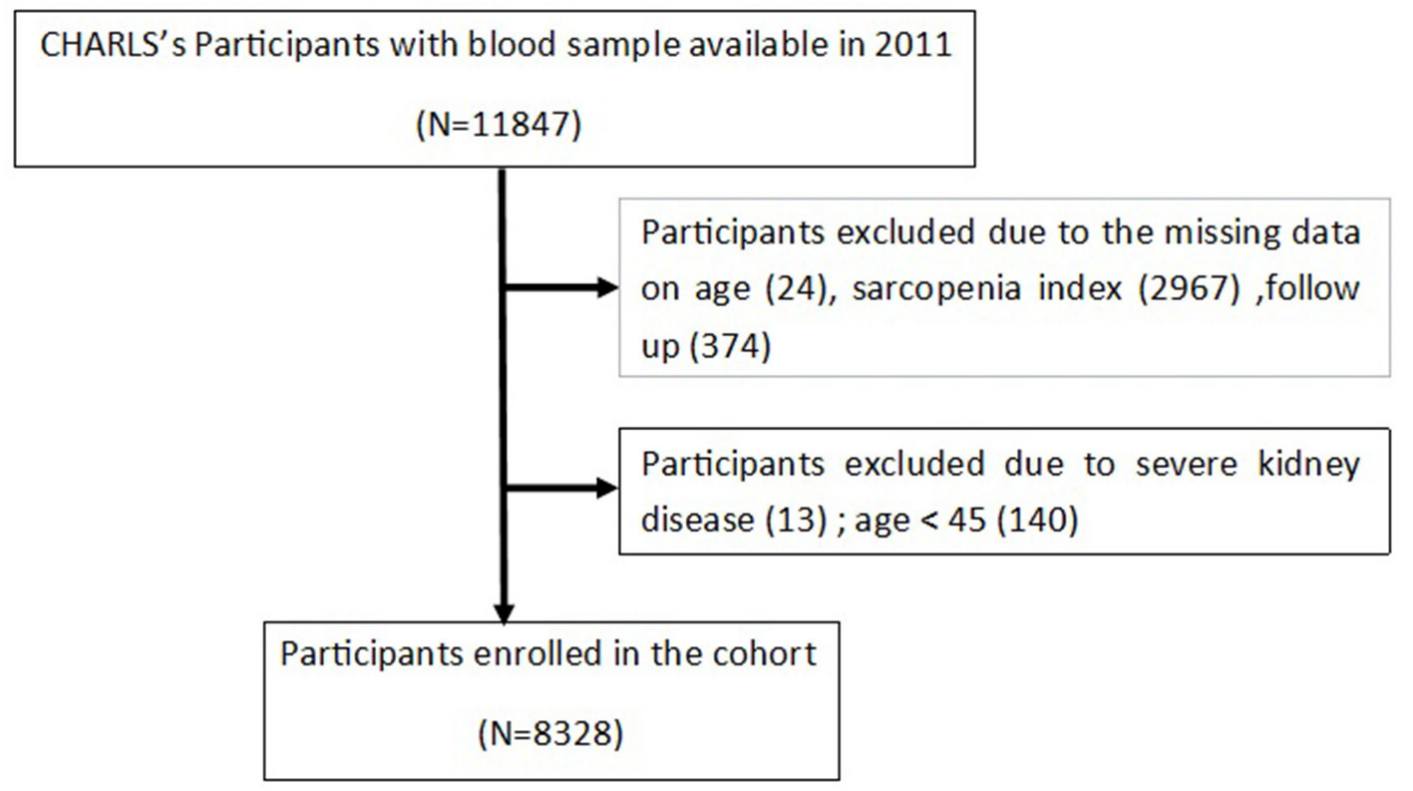
The flowchart of study participants.

Individuals: (1) aged not <45 years old at wave 1; (2) with complete information about serum creatinine and cystatin C in wave 1; (3) followed up at least one time in wave 2, 3, and 4 were included in the cohort. Individuals with eGFR < 30 ml/min/1.73 m^2^ were excluded from the study. A total of 8,328 individuals meeting these criteria were enrolled in the final cohort (Figure 1).

### All-cause mortality and onset time of death assessment

During the follow-up in wave 2, 3, and 4, the mortality of the participants was assessed by the interview status (alive or dead). While the exact date of death could be extracted from wave 2, there was little information about the exact date of death in wave 3 and 4. Once the death events happened, the survival time was calculated from the date of wave 1 to the death date in wave 2, or the median time from the first interview to the wave that records the death.

### Blood sample

The blood collection was executed by the staff of the Chinese Center for Disease Control and Prevention (China CDC) basing on the standard protocol. The fresh venous blood samples were transported at 4°C temperature and sent to the local laboratory, where the whole blood was collected to obtain plasma and buff coat. Then the plasma and buff coat were both frozen at −20°C, transported to Beijing within 2 weeks, and they would be placed in a deep freezer, and stored at −80°C until assay. The creatinine would be measured using the rate-blanked and compensated Jaffe creatinine method, while a particle-enhanced turbimetric assay was used to analyze the cystatin C. Other blood metrics, including Glucose, total cholesterol (TC), TG, low-density lipoprotein cholesterol (LDL-C), high-density lipoprotein cholesterol (HDL-C), Glycated hemoglobin (HbA1c), and so on, were also assayed.

### Other covariates

Based on the well-designed questionnaire, the CHARLS-trained interviewers collected information on demographic background, health status, and biomarkers. Demographic background including age, gender (male/female), education level (illiteracy, primary school, middle school, high school, and above), marriage status (married/along), Residence (urban/rural) were collected, Health status including fourteen comorbidities (hypertension, diabetes, dyslipidemia, cancer, kidney disease, stroke, heart problem, chronic lung disease, liver disease, digestive disease, nervous problem, memory-related diseases, arthritis, and asthma) diagnosed by doctor and treatment taken by respondents.

SI was calculated as (serum creatinine divided by serum Cystatin C) × 100. To give a more precise definition, hypertension was re-diagnosed as one of the following criteria: (1) an SBP higher than 140 mm Hg or a DBP higher than 90 mmHg; (2) self-report of a doctor's diagnosis; (3) self-report of the antihypertensive treatment. Diabetes was re-diagnosed as one of the following criteria: (1) self-report of a doctor diagnosis; (2) HbA1c ≥ 6.5%; (3) plasma glucose ≥ 7.0 mmol/L (fasting), or plasma glucose ≥ 11.1 mmol/L (casual); (4) self-report of the glucose-lowering or insulin treatment. Dyslipidemia was re-diagnosed as one of the following criteria: (1) self-report of a doctor diagnosis; (2) total cholesterol (TC) ≥ 240 mg/dL; (3) high-density lipoprotein cholesterol (HDL) ≤ 40 mg/dL; (4) low-density lipoprotein cholesterol (LDL) ≥ 160 mg/dL; (5) triglycerides (TG) ≥ 150 mg/dL; (6) self-report of the anti-dyslipidemia treatment. Kidney disease was re-diagnosed as one of the following criteria: (1) self-report of a doctor diagnosis; (2) self-report of the kidney disease treatment; (3) eGFR <90 ml/min/1.73m^2^. Estimated glomerular filtration rates were calculated using the CKD-EPI creatinine formula.

### Statistical analysis

Based on the quartile of SI, Participants were divided into four groups (Q1, 8.33–66.53; Q2, 66.53–76.84; Q3, 76.84–89.16; Q4, 89.16–274.43), with quartile 1 as the reference group. All variables were shown as follows: median (IQR) for continuous variables and counts with percentages for categorical variables. Mann-Whitney *U*-test and Fisher's exact test were used to assess balance in baseline characteristics among cohorts with different sarcopenia index levels. The survival curve was plotted with Kaplan-Meier analysis, and the difference in survival was tested by log-rank test. Univariate and multivariate Cox proportional-hazards regression model analyses were used. Variables that showed a significant relationship (*p* < 0.1) with the outcome were included in the step-wise multivariate analysis. Finally, three models were constructed, including a non-adjusted model, a minimally adjusted model (only adjusted for age and gender), and a fully adjusted model (adjusted for age, gender, BMI, education level, marriage status, hypertension, diabetes, eGFR, cancer, chronic lung disease, memory related disease, and smoking). The dose relationship between sarcopenia index and all-cause mortality was further assessed by the cubic spline functions and smooth curve fitting (penalized spline method). Subgroup analyses by gender (male vs. female), age (<60 vs. ≥60 years), BMI (<18.5; 18.5–24; ≥24), residence (rural vs. urban areas), combined with comorbidities or not (hypertension, diabetes, and dyslipidemia), eGFR (< 90; ≥90) were further performed to test the robustness of the results. As SI was calculated through two serum markers, both of which were used to assess the kidney function, Sensitivity analysis was utilized: (1) in participants without a history of cancer and eGFR > 60 ml/min/1.73 m^2^; (2) in participants without a history of cancer and eGFR > 90 ml/min/1.73 m^2^; (3) in the participants, without a history of kidney disease and cancer; to test the robustness of our findings.

Statistical analyses were performed using SPSS 26.0 (SPSS Inc., Chicago, IL, USA) and R package (version 4.2.1), *P* < 0.05 was considered statistically significant.

## Results

### Baseline characteristics of study participants

A total of 8,328 individuals were included in the study, of whom 934 (11.2%) died at follow-up. The cohort was categorized into four groups (Q1, Q2, Q3, Q4) based on the quartile of the sarcopenia index. The baseline characteristics are summarized in Table 1. The median age of the cohort was 59 years old, and males accounted for 47.3%. Compared to other groups, participants in Q4 were younger, had a higher proportion of males, with a higher educated level. Besides these, the mortality was 18.0, 11.3, 8.7, and 6.8% in quartiles 1–4.

**Table 1:** Baseline characteristics of the study participants (*N* = 8,328).

	**Overall**	**Q1**	**Q2**	**Q3**	**Q4**	
	***n*** **= 8,328**	***n*** **= 2,082**	***n*** **= 2,073**	***n*** **= 2,091**	***n*** **= 2,082**	***p***
Age	59.00 [53.00, 68.00]	64.00 [56.00, 73.00]	61.00 [54.00, 69.00]	58.00 [51.00, 65.00]	56.00 [50.00, 63.00]	< 0.001
Gender, *n* (%)						< 0.001
Female	4,386 (52.7)	1,523 (73.2)	1,248 (60.2)	954 (45.6)	661 (31.7)	
Male	3,942 (47.3)	559 (26.8)	825 (39.8)	1,137 (54.4)	1,421 (68.3)	
BMI	23.03 [20.70, 25.68]	22.54 [20.02, 25.38]	22.85 [20.54, 25.42]	23.21 [20.92, 25.70]	23.57 [21.38, 26.12]	< 0.001
Education level, *n* (%)						< 0.001
Illiteracy	4,075 (49.0)	1,311 (63.0)	1,115 (53.9)	937 (44.9)	712 (34.2)	
Primary school	1,862 (22.4)	413 (19.8)	441 (21.3)	475 (22.7)	533 (25.6)	
Middle school	1,543 (18.5)	237 (11.4)	341 (16.5)	438 (21.0)	527 (25.3)	
High school and above	841 (10.1)	120 (5.8)	173 (8.4)	239 (11.4)	309 (14.8)	
Residence, *n* (%)						< 0.001
Rural	6,869 (82.5)	1,827 (87.8)	1,742 (84.1)	1,697 (81.2)	1,603 (77.0)	
Urban	1,455 (17.5)	255 (12.2)	330 (15.9)	392 (18.8)	478 (23.0)	
Marriage status, *n* (%)						< 0.001
Unmarried	1,565 (18.8)	543 (26.1)	388 (18.7)	327 (15.6)	307 (14.7)	
Married	6,763 (81.2)	1,539 (73.9)	1,685 (81.3)	1,764 (84.4)	1,775 (85.3)	
Hypertension, *n* (%)	3,690 (44.5)	1,000 (48.2)	931 (45.2)	889 (42.7)	870 (42.0)	< 0.001
Diabetes, *n* (%)	1,245 (15.1)	268 (13.0)	281 (13.8)	317 (15.3)	379 (18.3)	< 0.001
Dyslipidemia, *n* (%)	4,014 (48.3)	857 (41.2)	579 (28.4)	988 (47.3)	857 (41.3)	< 0.001
Kidney disease, *n* (%)	842 (10.2)	190 (9.2)	189 (9.2)	224 (10.8)	239 (11.6)	0.022
Stroke, *n* (%)	208 (2.5)	59 (2.8)	55 (2.7)	49 (2.4)	45 (2.2)	0.502
eGFR (ml/min/1.73 m^2^)	93.00 [82.00, 101.00]	96.00 [87.00, 104.00]	94.00 [84.00, 102.00]	93.00 [82.00, 100.50]	89.00 [77.00, 98.00]	< 0.001
Cancer, *n* (%)	83 (1.0)	24 (1.2)	24 (1.2)	21 (1.0)	14 (0.7)	0.348
Chronic lung diseases, *n* (%)	904 (10.9)	252 (12.2)	224 (10.9)	221 (10.6)	207 (10.0)	0.158
Liver disease, *n* (%)	315 (3.8)	78 (3.8)	78 (3.8)	75 (3.6)	84 (4.1)	0.889
Heart problem, *n* (%)	1,069 (12.9)	295 (14.3)	283 (13.8)	253 (12.2)	238 (11.5)	0.025
Stomach or digestive disease, *n* (%)	1,939 (23.4)	487 (23.5)	498 (24.1)	496 (23.8)	458 (22.1)	0.444
Nervous problems, *n* (%)	127 (1.5)	42 (2.0)	38 (1.8)	27 (1.3)	20 (1.0)	0.02
Memory related disease, *n* (%)	119 (1.4)	42 (2.0)	31 (1.5)	24 (1.2)	22 (1.1)	0.04
Arthritis or Rheumatism, *n* (%)	2,954 (35.6)	839 (40.4)	734 (35.6)	704 (33.8)	677 (32.7)	< 0.001
Asthma, *n* (%)	320 (3.9)	108 (5.2)	82 (4.0)	66 (3.2)	64 (3.1)	0.001
Smoking, *n* (%)	3,293 (39.7)	584 (28.1)	739 (35.8)	930 (44.6)	1,040 (50.1)	< 0.001
Drinking, *n* (%)	2,661 (32.1)	660 (31.7)	650 (31.4)	677 (32.5)	674 (32.5)	0.828

BMI, Body mass index; eGFR, estimated glomerular filtration rate.

### Relation between sarcopenia index level and all-cause mortality

**Figure 2:**
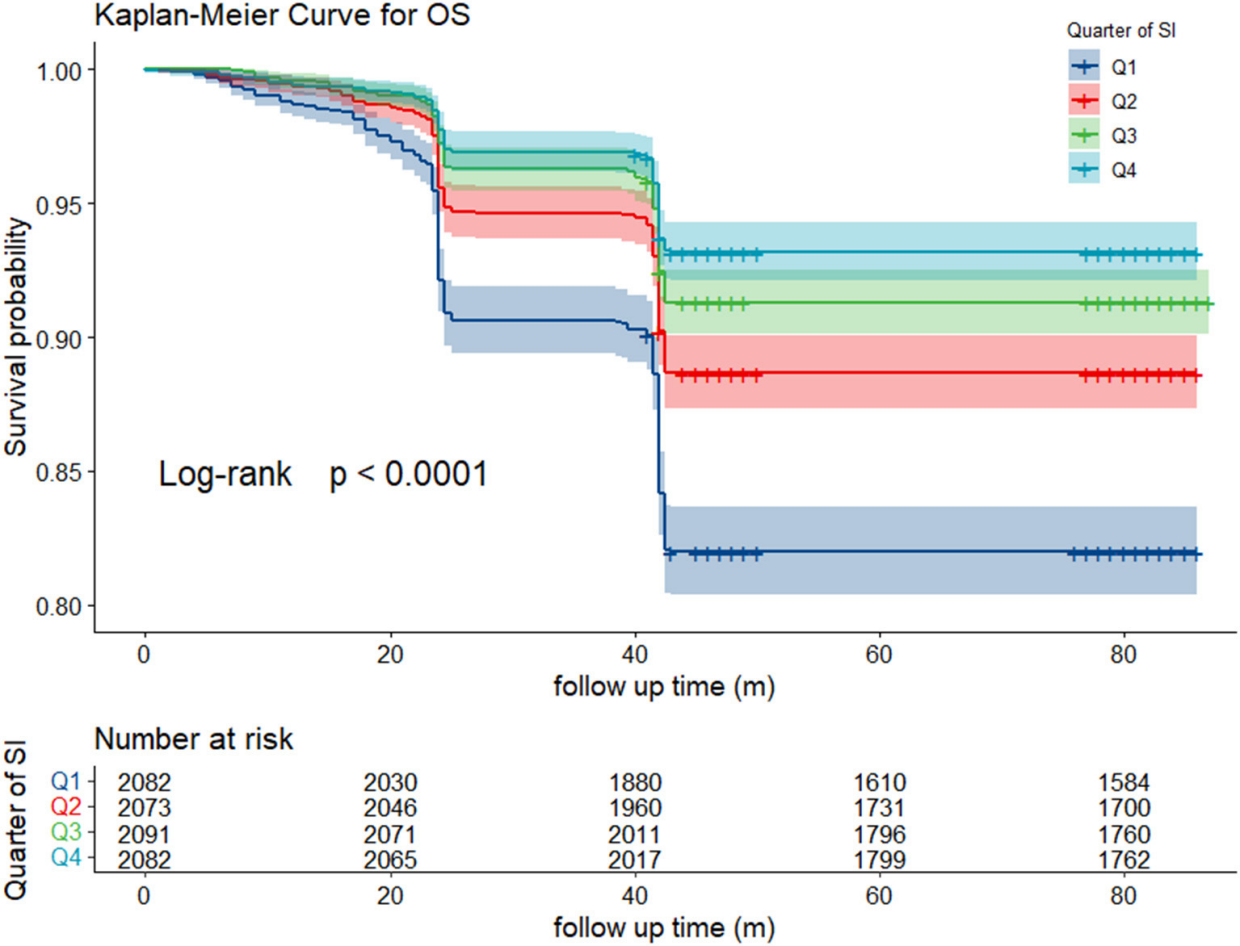
Kaplan-Meier curve of survival rate among different SI level.

**Figure 3:**
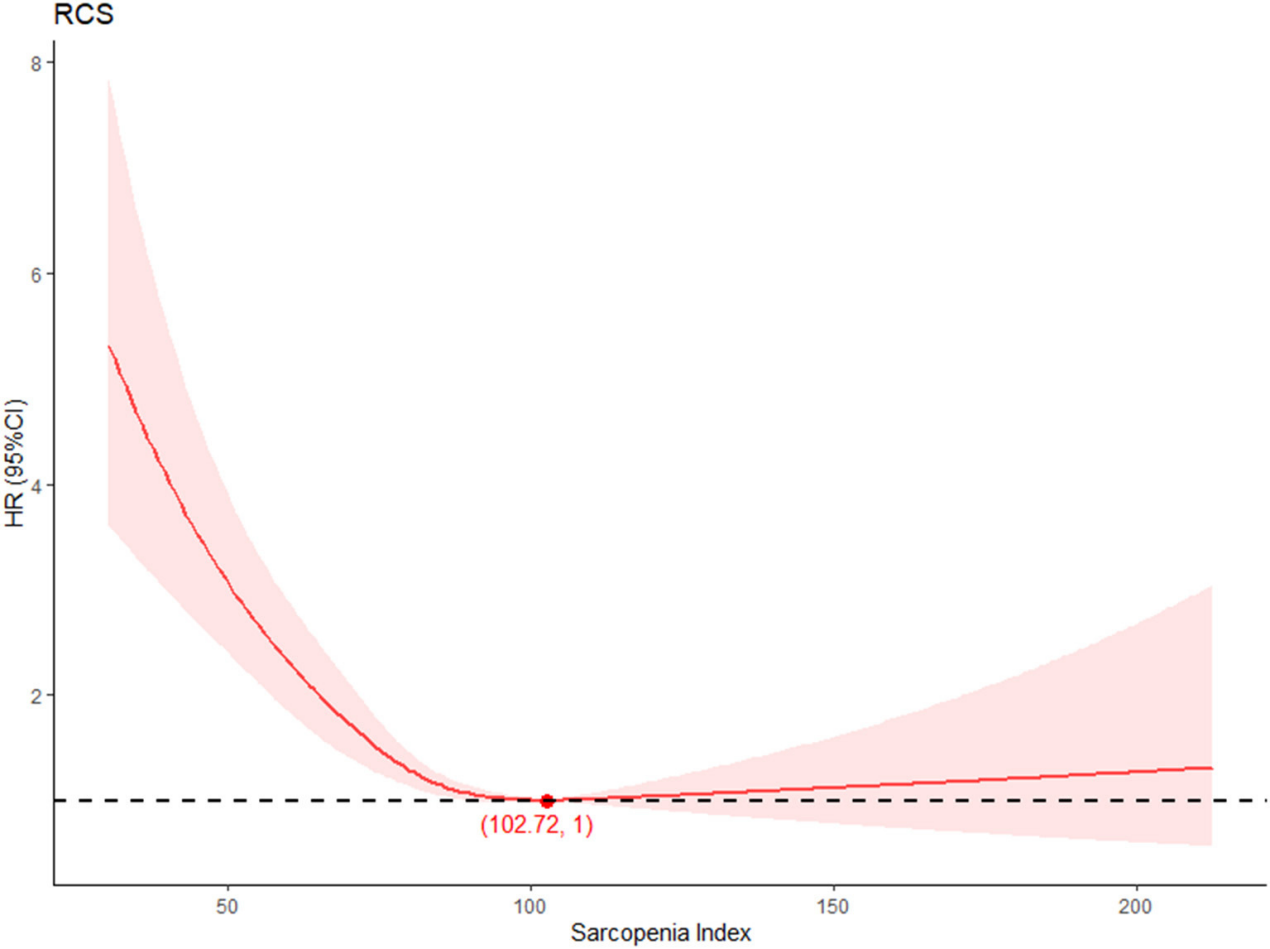
Dose-response relationship between SI level and all-cause mortality.

As is shown in Figure 2, participants with high SI had low mortality during follow-up. Significant differences in the all-cause mortality were shown by Kaplan–Meier curves (log-rank test, *p* < 0.001) among different SI levels. The relationship between SI and all-cause mortality was further assessed in the univariate and multivariate hazard ratio Cox regression model (Table 2). After adjusting for covariates, the SI showed a negative relationship with all-cause mortality (HR, 0.98; 95% CI, 0.98–0.99) in the cohort. The influence of different SI levels on all-cause mortality was further studied (Table 3). Q4 showed a significantly lower risk of death (HR, 0.36; 95% CI, 0.30–0.43; *p* for trend < 0.001), this correlation still exists after adjusting for multiple confounding factors. The restricted cubic spline model showed an L-shaped association between SI and all-cause mortality (Figure 3), with an infection point (SI = 102.72) after adjusting for multiple variables.

**Table 2:** Univariate and multivariate analysis the relationship between SI and all-cause mortality.

**Characteristics**	**Univariate**		**Multivariate**	
	**HR (95% CI)**	***P***	**HR (95% CI)**	***P***
Sarcopenia index	0.979 (0.975–0.982)	<0.001	0.983 (0.977–0.988)	<0.001
Age	1.100 (1.09–1.11)	<0.001	1.059 (1.048–1.071)	<0.001
Gender	1.660 (1.46–1.89)	<0.001	1.803 (1.457–2.232)	<0.001
BMI	0.895 (0.88–0.91)	<0.001	0.959 (0.939–0.980)	<0.001
Education level	0.686 (0.64–0.74)	<0.001	0.869 (0.793–0.953)	0.003
Residence	0.784 (0.65–0.94)	0.009		
Marriage status	0.490 (0.43–0.56)	<0.001	0.824 (0.695–0.978)	0.026
Hypertension	1.725 (1.51–1.96)	<0.001	1.258 (1.078–1.468)	0.004
Diabetes	1.490 (1.27–1.75)	<0.001	1.446 (1.199–1.745)	<0.001
Dyslipidemia	0.942 (0.83–1.07)	0.058		
Kidney disease	2.629 (2.29–3.01)	<0.001		
Stroke	2.735 (2.09–3.58)	<0.001		
eGFR	0.966 (0.96–0.97)	<0.001	0.984 (0.978–0.99)	<0.001
Cancer	1.752 (1.05–2.92)	0.031	2.522 (1.454–4.374)	0.001
Chronic lung diseases	2.003 (1.70–2.36)	<0.001	1.458 (1.211–1.756)	<0.001
Liver disease	1.243 (0.92–1.69)	0.163		
Heart problem	1.287 (1.08–1.54)	0.005		
Stomach or digestive disease	0.825 (0.70–0.97)	0.018		
Nervous problems	1.548 (1.00–2.39)	0.048		
Memory related disease	3.166 (2.27–4.42)	<0.001	1.664 (1.096–2.529)	0.017
Arthritis or rheumatism	0.924 (0.81–1.06)	0.252		
Asthma	2.002 (1.56–2.57)	<0.001		
Smoking	1.673 (1.47–1.9)	<0.001	1.302 (1.073–1.579)	0.007
Drinking	0.906 (0.79–1.04)	0.165		

HR, hazard ratio; CI, confidence interval; BMI, Body mass index; eGFR, estimated glomerular filtration rate.

**Table 3:** Association between SI and all-cause mortality in different model.

**Quintiles of sarcopenia index**		**No. of events/No. of participants**	**Model 1**		**Model 2**		**Model 3**		**Non-linear *p*-value**
			**HR (95% CI)**	***p***	**HR (95% CI)**	***p***	**HR (95% CI)**	***p***	
Sarcopenia index (continue)			0.98 (0.97–0.98)	<0.001	0.99 (0.98–0.99)	<0.001	0.98 (0.98–0.99)	<0.001	<0.001
Sarcopenia index (quartile)									
	Q1	375/2,082	Ref.	-	Ref.	-	Ref.	-	
	Q2	235/2,073	0.61 (0.52–0.71)	<0.001	0.70 (0.59–0.83)	<0.001	0.63 (0.52–0.76)	<0.001	
	Q3	182/2,091	0.46 (0.39–0.55)	<0.001	0.61 (0.51–0.74)	<0.001	0.50 (0.40–0.63)	<0.001	
	Q4	142/2,082	0.36 (0.30–0.43)	<0.001	0.55 (0.45–0.68)	<0.001	0.44 (0.34–0.57)	<0.001	
	*P* for trend		-	<0.001	-	<0.001	-	<0.001	

HR, hazard ratio; CI, confidence interval.

Model 2: adjusted by age and gender.

Model 3: adjusted by age, gender, BMI, education level, marriage status, hypertension, diabetes, eGFR, cancer, chronic lung disease, memory related disease, and smoking.

### Subgroup analyses and sensitive analysis

Subgroup analysis was utilized to clarify the relationship between different levels of SI and all-cause mortality by potential risk factors. As is shown in Table 4, participants with high SI levels showed decreased risk in all-cause mortality among all the analyses. However, as SI was calculated through serum creatinine and cystatin C, both of which are important markers in evaluating kidney function, sensitivity analysis was conducted. The results were consistent with previous analysis (Supplementary Tables 1–3).

**Table 4:** Subgroup analysis of HRs (95% CIs) of SI for all-cause mortality.

	**Q1**	**Q2**	**Q3**	**Q4**	***P*** **for trend**	***P*** **for interaction**
	**HR (95% CI)**	**HR (95% CI)**	**HR (95% CI)**	**HR (95% CI)**		
Gender						0.291
Male	1.00 (Ref)	0.66 (0.51–0.85)	0.50 (0.38–0.66)	0.43 (0.31–0.59)	<0.001	
Female	1.00 (Ref)	0.57 (0.42–0.77)	0.49 (0.33–0.73)	0.44 (0.28–0.71)	<0.001	
Age						0.872
Age < 60	1.00 (Ref)	0.52 (0.30–0.91)	0.55 (0.33–0.93)	0.34 (0.18–0.61)	0.001	
Age ≥ 60	1.00 (Ref)	0.55 (0.45–0.67)	0.36 (0.28–0.45)	0.32 (0.24–0.42)	<0.001	
BMI						0.064
< 18.5	1.00 (Ref)	0.61 (0.39–0.97)	0.37 (0.19–0.69)	0.18 (0.06–0.61)	<0.001	
18.5–24	1.00 (Ref)	0.62 (0.48–0.80)	0.55 (0.41–0.75)	0.56 (0.40–0.78)	<0.001	
≥24	1.00 (Ref)	0.68 (0.47–1.00)	0.48 (0.31–0.74)	0.34 (0.21–0.56)	<0.001	
Residence						1
Urban	1.00 (Ref)	0.40 (0.23–0.69)	0.31 (0.17–0.56)	0.24 (0.12–0.46)	<0.001	
Rural	1.00 (Ref)	0.67 (0.55–0.82)	0.54 (0.43–0.69)	0.48 (0.37–0.64)	<0.001	
Hypertension						0.008
With	1.00 (Ref)	0.58 (0.45–0.74)	0.45 (0.33–0.60)	0.48 (0.35–0.66)	<0.001	
Without	1.00 (Ref)	0.72 (0.53–0.97)	0.62 (0.44–0.87)	0.42 (0.27–0.64)	<0.001	
Diabetes						0.103
With	1.00 (Ref)	0.54 (0.34–0.86)	0.48 (0.29–0.80)	0.46 (0.28–0.77)	0.006	
Without	1.00 (Ref)	0.62 (0.50–0.77)	0.50 (0.39–0.64)	0.43 (0.32–0.57)	<0.001	
Dyslipidemia						0.12
With	1.00 (Ref)	0.74 (0.55–0.98)	0.54 (0.39–0.75)	0.51 (0.36–0.73)	<0.001	
Without	1.00 (Ref)	0.54 (0.42–0.70)	0.48 (0.36–0.66)	0.39 (0.27–0.56)	<0.001	
eGFR						0.548
eGFR < 90	1.00 (Ref)	0.64 (0.50–0.82)	0.46 (0.35–0.61)	0.50 (0.37–0.67)	<0.001	
eGFR ≥ 90	1.00 (Ref)	0.67 (0.49–0.91)	0.79 (0.56–1.12)	0.54 (0.35–0.84)	0.008	

BMI, Body mass index; eGFR, estimated glomerular filtration rate.

In the multivariate models, confounding factors such as age, gender, BMI, education level, marriage status, hypertension, diabetes, eGFR, cancer, chronic lung disease, memory related disease, and smoking were included unless the variable was used as a subgroup variable.

## Discussion

Though the correlation between SI and all-cause mortality was studied in several studies, our study was the first to evaluate this relationship in middle-aged and older adults Chinese. As the result shows, SI level showed a positive relationship with survival rate. Differences in survival rate were further compared among different levels of SI. Participants with the lowest SI level showed the highest mortality compared to other groups, which was after multivariate adjustment. Among the cohort, patients with higher SI had lower mortality, meaning SI could be an effective marker for assessing mortality in middle-aged and older adults Chinese.

Serum creatinine (SCr) and cystatin C (CysC) is serum markers used to evaluate the glomerular filtration rate (GFR) and renal function (11, 12). As the SCr was mainly generated in muscle from phosphocreatine and creatine, the amount of skeletal muscle mass had a profound impact on SCr secreted into circulation. The concentration of SCr is often maintained as the muscle mass is stable. However, such factors, including aging, gender, chronic illness, and augmented renal clearance, could lead to a fluctuation in SCr (13, 14). Though there were some studies clarifying the relationship between low SCr and adverse events (6, 13, 15, 16), the fluctuation of SCr often limits the wide usage of Scr. CysC, which is metabolized by the proximal tubular cells, is excreted by all nucleated cells. CysC often keeps at a stable level without any fluctuation, leading to a conclusion that the impact of muscle mass on CysC is significantly < SCr. Considering the characteristic of SCr and CysC, SI was developed by (SCr / Cysc) ^*^100 to assess sarcopenia (17). Our study shows the relationship between low SI and all-cause mortality. Patients with low SI often suffer from losing SMM. Patients with a low level of SMM often suffer from different kinds of diseases. Many studies indicated the relationship between SMM loss and reduction of life quality. For the correlation between SI and SMM, it is not surprising to make a conclusion that low SI could be a good marker of predicting the patients' prognosis.

As SI was developed in recent years, there were several studies evaluating the relationship between SI and all-cause mortality. In a retrospective study shown by Kashani et al. (6), 226 high-risk adults in ICU were recruited, and lower SI was a risk factor for in-hospital and 90-day mortality. Tang et al. explored the relationship in hospitalized older patients (9), SI showed a significant association with all-cause mortality after 3-year follow-up. Ren et al. further investigated this in a prospective study (8). After the median follow-up period of 212 days, the SI showed a close relationship with long-term mortality, malnutrition, and sarcopenia in older Chinese patients. Our results were in consists with these previous studies, which indicated the predictive effect of SI on all-cause mortality. There were also some studies exploring the relationship between SI and adverse events in clinical patients. According to the study held by Suzuki et al. (12), SI was correlated with chemotherapy-related adverse effects in patients diagnosed with lung cancer. A multicenter prospective study held by Komorita et al. showed a close relationship between lower SI and fractures in patients with T2DM (18). Among these studies, participants with abnormal kidney dysfunction were excluded. To further testify to the effectiveness of our results, sensitivity analysis and subgroup analysis were both put into practice, both of which were consistent with the previous conclusion. Though the accuracy of SI in predicting sarcopenia was confirmed in a previous study, other studies argued that the relationship between SI and SMM was debatable (19), and needed further study. For the limited information given by CHARLS database, the relationship between SI and sarcopenia was not testified in this study. However, for the promise of SI in predicting adverse events, further prospective study was needed to confirm the relationship between SI and sarcopenia and adverse events.

One major strength of our study was that this was the most significant cohort enrolled to analyze the relationship between sarcopenia index and all-cause mortality. Besides this, this study also brought the sarcopenia index more clinically possess value. However, there were still some limitations that should be noticed. First, due to the details collected by CHARLS, most of the exact death date was ambiguous during the research, which unavoidable brought bias to the survival analysis. Second, all the health information, including the chronic disease, was collected based on the self-report by participants. However, some participants might be unaware of their diseases. Multiple measures, including laboratory tests and treatment messages, were all collected to alleviate this bias. Third, all the data used in this study originated from the CHARLS, which was a representative national middle-aged and older adults database in China. It was unclear whether the conclusion could be applied to other countries.

## Conclusion

This study explored the relationship between SI and all-cause mortality among middle-aged and older adults Chinese. As the result shows, low SI was closely related to elevated mortality. More attention should be paid to individuals with low SI. The sensitivity analysis also confirmed the relationship.

## Data availability statement

The raw data supporting the conclusions of this article will be made available by the authors, without undue reservation.

## Ethics statement

The studies involving human participants were reviewed and approved by Biomedical Ethics Review Committee of Peking University (IRB00001052-11015). The Ethics Committee waived the requirement of written informed consent for participation.

## Author contributions

YW, HW, and YT: methodology, writing, and revision. XZ, YL, QL, and JR: data curation and investigation. CL: supervision, reviewing, and editing the manuscript. All authors contributed to the article and approved the submitted version.
