# Supplementary material for: Sarcopenia index based on serum creatinine and cystatin C is associated with mortality in middle-aged and older adults in Chinese: A retrospective cohort study from the China Health and Retirement Longitudinal Study
Source: Front Public Health. 2023 Mar 21;11:1122922. doi: 10.3389/fpubh.2023.1122922 (PMC10071508; doi:10.3389/fpubh.2023.1122922)
Supplement: Supplementary file 1 [file Table_1.docx]

| Supplement table 1: Sensitivity analysis excluded individuals diagnosed with cancer or eGFR <60ml/min/1.73^2 (N=462) for association of SI levels and with all-cause mortality in CAHRLS (2011-2018) | | | | | | | | | |
| --- | --- | --- | --- | --- | --- | --- | --- | --- | --- |
| Quintiles of sarcopenia index | No. of events/No. of participants | Model 1 | |  | Model 2 | |  | Model 3 | |
|  |  | HR (95% CI) | p |  | HR (95% CI) | p |  | HR (95% CI) | p |
| Q1 | 338/1973 | 1 (Ref.) |  |  | 1 (Ref.) |  |  | 1 (Ref.) |  |
| Q2 | 204/1975 | 0.58 (0.49-0.69) | <0.001 |  | 0.66 (0.55-0.79) | <0.001 |  | 0.61 (0.50-0.75) | 0.024 |
| Q3 | 153/1972 | 0.43 (0.36-0.52) | <0.001 |  | 0.59 (0.48-0.72) | <0.001 |  | 0.53 (0.42-0.68) | 0.200 |
| Q4 | 116/1946 | 0.33 (0.27-0.41) | <0.001 |  | 0.53 (0.42-0.67) | <0.001 |  | 0.48 (0.36-0.64) | 0.014 |
| P for trend |  | - | <0.001 |  | - | <0.001 |  | - | 0.016 |
| Abbreviations: HR, hazard ratio; CI, confidence interval; Model 2: adjusted by age and gender Model 3: adjusted by age, gender, BMI, education level, marriage status, hypertension, diabetes, chronic lung disease, memory related disease and smoking | | | | | | | | | |
|  |  |  |  |  |  |  |  |  |  |
