# Supplementary material for: Sarcopenia index based on serum creatinine and cystatin C is associated with mortality in middle-aged and older adults in Chinese: A retrospective cohort study from the China Health and Retirement Longitudinal Study
Source: Front Public Health. 2023 Mar 21;11:1122922. doi: 10.3389/fpubh.2023.1122922 (PMC10071508; doi:10.3389/fpubh.2023.1122922)
Supplement: Supplementary file 2 [file Table_2.docx]

| Supplement table 2: Sensitivity analysis excluded individuals diagnosed with cancer or eGFR <90ml/min/1.73^2 (N=3463) for association of SI levels and with all-cause mortality in CAHRLS (2011-2018) | | | | | | | | | |
| --- | --- | --- | --- | --- | --- | --- | --- | --- | --- |
| Quintiles of sarcopenia index | No. of events/No. of participants | Model 1 | |  | Model 2 | |  | Model 3 | |
|  |  | HR (95% CI) | p |  | HR (95% CI) | p |  | HR (95% CI) | p |
| Q1 | 147/1368 | Ref. |  |  | Ref. |  |  | Ref. |  |
| Q2 | 77/1280 | 0.55 (0.42-0.72) | <0.001 |  | 0.56 (0.42-0.74) | <0.001 |  | 0.69 (0.51-0.95) | 0.024 |
| Q3 | 68/1216 | 0.51 (0.38-0.68) | <0.001 |  | 0.53 (0.39-0.71) | <0.001 |  | 0.79 (0.56-1.13) | 0.200 |
| Q4 | 35/1001 | 0.31 (0.22-0.46) | <0.001 |  | 0.33 (0.23-0.49) | <0.001 |  | 0.57 (0.37-0.89) | 0.014 |
| P for trend |  | - | <0.001 |  | - | <0.001 |  | - | 0.016 |
| Abbreviations: HR, hazard ratio; CI, confidence interval; Model 2: adjusted by age and gender Model 3: adjusted by age, gender, BMI, education level, marriage status, hypertension, diabetes, chronic lung disease, memory related disease and smoking | | | | | | | | | |
|  |  |  |  |  |  |  |  |  |  |
