# Supplementary material for: Sarcopenia index based on serum creatinine and cystatin C is associated with mortality in middle-aged and older adults in Chinese: A retrospective cohort study from the China Health and Retirement Longitudinal Study
Source: Front Public Health. 2023 Mar 21;11:1122922. doi: 10.3389/fpubh.2023.1122922 (PMC10071508; doi:10.3389/fpubh.2023.1122922)
Supplement: Supplementary file 3 [file Table_3.docx]

| Supplement table 3: Sensitivity analysis excluded individuals diagnosed with cancer or kidney disease (N=3823) for association of sarcopenia index levels and with all-cause mortality in CAHRLS (2011-2018) | | | | | | | | | |
| --- | --- | --- | --- | --- | --- | --- | --- | --- | --- |
| Quintiles of sarcopenia index | No. of events/No. of participants | Model 1 | |  | Model 2 | |  | Model 3 | |
|  |  | HR (95% CI) | p |  | HR (95% CI) | p |  | HR (95% CI) | p |
| Q1 | 136/1279 | Ref. |  |  | Ref. |  |  | Ref. |  |
| Q2 | 72/1201 | 0.55 (0.41-0.73) | <0.001 |  | 0.56 (0.42-0.75) | <0.001 |  | 0.69 (0.50-0.96) | 0.027 |
| Q3 | 63/1131 | 0.51 (0.38-0.69) | <0.001 |  | 0.54 (0.40-0.74) | <0.001 |  | 0.80 (0.56-1.15) | 0.228 |
| Q4 | 31/894 | 0.30 (0.21-0.45) | <0.001 |  | 0.32 (0.21-0.49) | <0.001 |  | 0.57 (0.36-0.91) | 0.019 |
| P for trend |  |  | <0.001 |  |  | <0.001 |  |  | 0.02 |
| Abbreviations: HR, hazard ratio; CI, confidence interval; Model 2: adjusted by age and gender Model 3: adjusted by age, gender, BMI, education level, marriage status, hypertension, diabetes, chronic lung disease, memory related disease and smoking | | | | | | | | | |
|  |  |  |  |  |  |  |  |  |  |
